# Titles and Semantic Violations Affect Eye Movements When Viewing Contemporary Paintings

**DOI:** 10.3389/fnhum.2022.808330

**Published:** 2022-03-04

**Authors:** Joanna Ganczarek, Karolina Pietras, Anna Stolińska, Magdalena Szubielska

**Affiliations:** ^1^Institute of Psychology, Pedagogical University of Cracow, Kraków, Poland; ^2^Institute of Computer Science, Pedagogical University of Cracow, Kraków, Poland; ^3^Institute of Psychology, The John Paul II Catholic University of Lublin, Lublin, Poland

**Keywords:** titles, semantic violations, eye movements, paintings, aesthetic judgements

## Abstract

The role of titles in perception of visual art is a topic of interesting discussions that brings together artists, curators, and researchers. Titles provide contextual cues and guide perception. They can be particularly useful when paintings include semantic violations that make them challenging for viewers, especially viewers lacking expert knowledge. The aim of this study is to investigate the effects of titles and semantic violations on eye movements. A total of 127 participants without expertise in visual art viewed 40 paintings with and without semantic violations (20 each) in one of three conditions: untitled, consistent titles and inconsistent titles. After each painting was viewed participants also rated liking and understanding. Our results suggest that titles affect the way paintings are viewed: both titled conditions were associated with shorter first fixation duration, longer saccade durations, and amplitudes and higher dynamic entropy than the untitled conditions. Titles were fixated on more frequently (but only in the time window between 1,200 and 2,800 ms) when presented alongside paintings with semantic violations than paintings without violations, and the percentage of fixations to titles was particularly high in the case of paintings with double inconsistencies (inconsistent titles and semantic violations). Also, we found that semantic violations attracted attention early on (300–900 ms), whereas titles received attention later (average first fixation on title was at 936.28 ms) and inconsistencies in titles were processed even later (after 4,000 ms). Finally, semantic violations were associated with higher dynamic entropy than paintings without violations. Our results demonstrate the importance of titles for processing of artworks, especially artworks that present a challenge for the viewers.

## Introduction

Titles are an important part of artworks and artists pay great attention to titles that their works are presented under (Gombrich, 1985; Welchman, 1997). Titles allow artists to communicate beyond the visual layer, help collectors and owners to catalog works, and guide audience experience. In the seventeenth and eighteenth centuries titles were often explicative and explanatory when a painting’s subject was little known. Also, they were descriptive and narrative describing a painting’s content as precisely as possible (Loibl, 2018). According to Welchman the turning point in titling was the Modernism (1870–1920) when other strategies of titling had been consolidated. Titles gained greater meaning and importance in art which is reflected by Duchamp’s statement that titles are the “invisible colors” (Welchman, 1997). Indeed, modern and contemporary art often plays on titles by providing (mis) information. Sometimes artworks are accompanied by explanatory titles aimed to guide interpretation. As in the case of Brancusi’s *Bird in Space*, the title allows viewers to see past polished materials and perceive the shapes in motion, in flight (Gombrich, 1985). Other times, however, titles are non-explanatory and purposely introduce inconsistencies. For example, Francis Picabia’s *Undie, jeune fille americaine* contains an unintelligible name “Undie,” adding complexity and ambiguity (Gombrich, 1985). Apart from distinction between explanatory and non-explanatory, many typologies of titles were proposed (e.g., Levinson, 1985; Welchman, 1997). For example, Welchman (1997) lists denotative, connotative titles, and the untitling condition. Denotative titles include words that are directly linked to what is represented in a painting (e.g., Kandinsky’s *Painting with a White Border*). Connotative titles are instead ambiguous and do not denote objects depicted. For example, in Duchamp’s *The Bride Stripped Bare by Her Bachelors, Even* or *The Large Glass*, this connotative title does not directly name objects in the artwork. Finally, the untitling category contains the *Untitled* titles and numeric titles which are a statement about the act of titling visual artworks, maybe even a rejection of titling (Welchman, 1997). Only the denotative, descriptive titles inform viewers about artworks. Both the connotative and untitled conditions may provide a hint of a meaning, but elude unambiguous solutions. Other author, Levinson (1985) lists nine different categories of titles such as neutral titles (naming objects represented in paintings), underlining titles (stressing a core subject of a painting), focusing titles (suggesting which subjects should in focus), undermining, ironic titles (suggesting an ironic interpretation), undermining, incongruous titles (playing with contradictions without irony), mystifying titles (changing perspective), disambiguating titles (specifying one content over another), allusive titles (referring to other work, artists etc.), and again interpretation (*Untitled*, numerical or purely descriptive).

In addition to these theoretical considerations, there is an ongoing discussion among art curators whether titles and other types of information about works are useful in guiding the audience’s experience of art (Pekarik, 2004) or whether artworks should be “self-explanatory” in their visual layer. The studies of art-interested audiences showed that in the case of visiting an exhibition of modern and contemporary art, viewers had no complaints about the lack of labels (Pekarik, 2004), and that short labels containing artists’ names, artwork title, technique, year of creation did not change the aesthetic judgments (Szubielska and Sztorc, 2019). Thus, perhaps in the situation of the reception of works of art in a museum or gallery by expert viewers, it is correct to say that “because they had not come for information, they did not miss it” (Pekarik, 2004, p. 14). The situation seems to be different for non-experts, who need interpretative hints, especially when dealing with challenging contemporary art (as suggested, for example, by Leder et al., 2004). Therefore, they spend time, whether viewing works of art in a gallery or a laboratory setting, not only looking at the art, but also labels (Brieber et al., 2014), especially when it comes to more ambiguous, abstract art (Szubielska et al., 2021c). When provided with original titles that adequately described the content of the painting, non-expert participants rated perceived meaningfulness higher and found paintings more understandable than participants who did not know artwork titles (Russell and Milne, 1997; Swami, 2013). At the same time, knowing descriptive titles did not change the rating of the images on scales of pleasingness, interestingness and complexity (Russell and Milne, 1997). In contrast, knowledge of the original, rather metaphorical titles (that did not provide a clear hint as to how to interpret the work) did not alter either the emotions or aesthetic judgments of naïve viewers viewing contemporary installations (Szubielska et al., 2021b). Furthermore, Belke et al. (2010) and Gerger and Leder (2015) testing the effect of semantic matching between title and painting found that non-experts liked images more when accompanied by a title relevant to the visual content (aptly describing the painting) than one unrelated to the content. Summing up, the positive effect of descriptive titles semantically consistent with the painting on liking and understanding, compared to the no-title condition, has been shown in some studies (Russell, 2003; Swami, 2013; Bubić et al., 2017), but not in others (Millis, 2001; Jucker et al., 2014).

**FIGURE 1:**
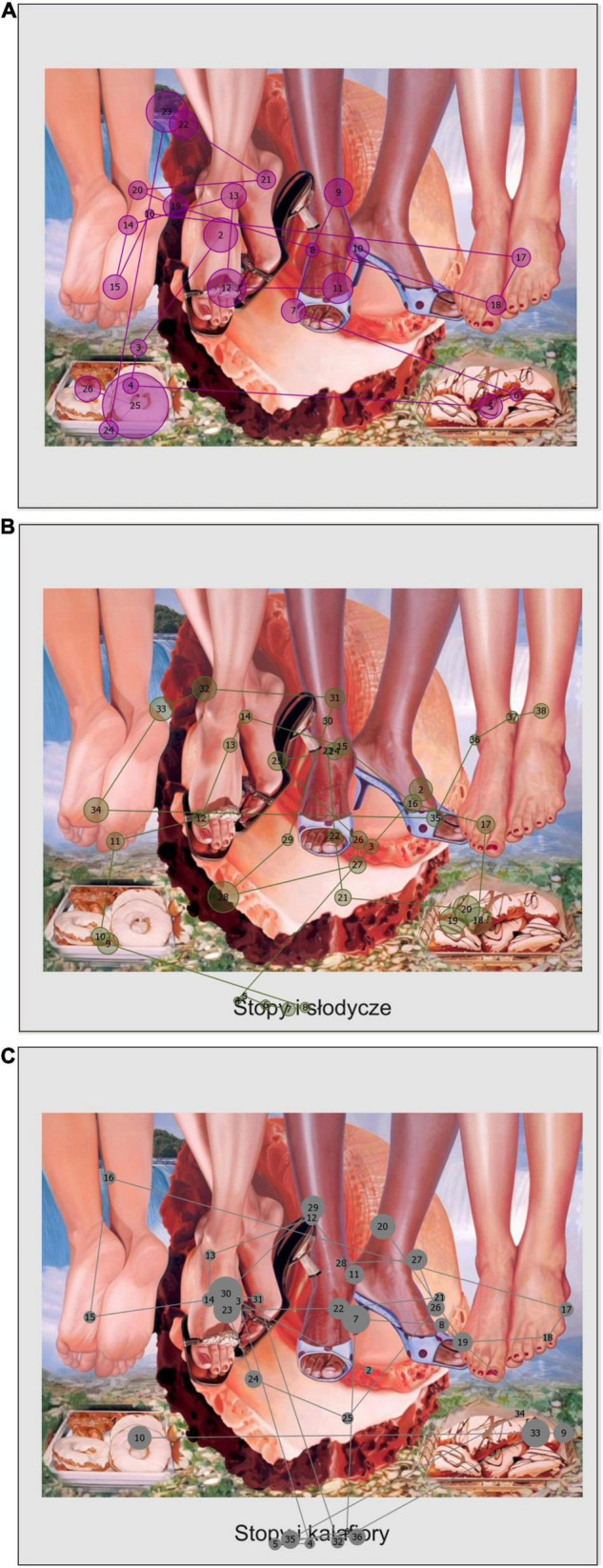
Examples of scanpaths from selected participants for a painting with semantic violations in three conditions. **(A)** Untitled, **(B)** consistent title (English translation: Feet and sweets), **(C)** inconsistent title (English translation: Feet and cauliflowers). The circles represent fixations, solid lines represent saccades, and the numbers correspond to indexes of fixations. The size of circles depicts relative fixation duration. Painting by Koons (2000), oil on canvas, 299.7 × 431.8 cm. Solomon R. Guggenheim Museum, New York. Commissioned by Deutsche Bank AG in consultation with the Solomon R. Guggenheim Foundation for the Deutsche Guggenheim, Berlin.

Moreover, the effects of titles may not only depend on the semantic (in) consistency between titles and paintings, but also inconsistencies in a painting itself. As a matter of fact, ambiguity is often considered to be the inherent value of an artwork (Zeki, 2003). Mysterious smile of *Mona Lisa* or polysemic gaze of *Girl with a Pearl* are only some well-known examples of how great masters create the puzzle which incites viewers to different interpretations. Ambiguity is particularly important in modern and postmodern art, which transgresses the paradigm of *mimesis*. Moreover, contemporary art introduces disfluency as a method of expression in which various forms of uncertainty, indeterminacy or strangeness are expected and appreciated (Bullot and Rolf, 2013). Art scientists are trying to capture some aspects of this complex phenomenon, appreciating the fact that ambiguity is a broad category, by using terms as semantic instability (Muth and Carbon, 2016), ambiguity (Jakesch et al., 2017), semantic inconsistencies (Markey et al., 2019) or semantic violations (Pietras and Ganczarek, 2018; Ganczarek et al., 2020; Szubielska et al., 2021a) to address the challenge which artworks present to viewers. In the present study we use the latter term, i.e., semantic violations to name a case in which a visual scene contains atypical objects (with no reference to the global meaning of the scene) or there are unusual relationships between objects in the scene. As in Jeff Koons’ work *Niagara* (see Figure 1), women’s feet are placed next to sweets and the Niagara Falls in the background. This juxtaposition of objects creates unusual relations between them, forming a collage of recognizable, but unrelated items.

Semantic violations defined in a similar way were first studied in the context of everyday life scenes. Some studies report that such violations attract attention (e.g., Loftus and Mackworth, 1978; Underwood et al., 2007), whilst others do not (e.g., Henderson et al., 1999; Võ and Henderson, 2009, 2011). In a series of interesting experiments, Võ and Henderson (2009) showed that indeed violations attract attention, but only once they are fixated. In such a case, semantically inconsistent objects are related to increased gaze durations and number of fixations. At the same time, they found no evidence of extrafoveal detection of semantic violations. Due to the fact that such semantic violations defy viewers’ expectations about where certain objects appear, these violations are related to longer latencies to fixations on target and consequently poor search performance (Võ and Henderson, 2011). The results of the latter study suggest that semantic violations are not detected during the initial 250 ms of viewing, but this initial quick glimpse helps viewers to recognize a scene and guides further search. Consequently, target objects in semantically non-violated scenes are fixated approximately after 1,255 ms compared to 1,723 ms for targets in semantically violated scenes. Taken together these results suggest that a general semantic category is detected very early (250 ms) and guides subsequent processing (e.g., Oliva and Torralba, 2006; Castelhano and Henderson, 2008). This contextual guidance is disturbed when a scene contains objects undergoing semantic violations. The early, albeit after the initial 250 ms, effect of semantic violations was shown in a neuroimaging study, where semantic violations elicited negative ERP responses in N300 (250–350 ms) and N400 (350–600 ms) time windows (Võ and Wolfe, 2013). The authors argue that the N300 component may reflect initial difficulties in perceptual processing and object identification, whereas the N400 may refer to increased post identification processing. Interestingly, a later effect of semantic violations was shown in the case of artworks, where semantic inconsistencies elicited a higher amplitude in the P600 (600–1,000 ms; Markey et al., 2019). Therefore, semantic violations were related to an intense processing, but it occurred later than in real-life scenes. This leads to the conclusion that semantic violations in paintings might be perceived differently than in regular life probably due to specific expectations viewers formulate toward art (i.e., people expect rules to be violated; Markey et al., 2019). Moreover, art might create a safe environment to experience ambiguity with positive affect, contrary to real life situations, in which ambiguity is often perceived as more threatening (see Jakesch et al., 2017). In fact, it seems that ambiguity in art may be expected and guide perception thus causing intense processing of semantic violations that are often appreciated by the viewers (e.g., Muth and Carbon, 2013; Jakesch et al., 2017; Ganczarek et al., 2020, but see Szubielska et al., 2021a).

Besides the effects of semantic violations, physiological parameters including eye movements studied here, can be influenced by the presence of textual information accompanying paintings. The studies investigating the effects of such information on eye movements when viewing contemporary art mostly focus on two sources of information: labels and titles. Research on the effects of long labels including explanatory texts report that the attention is similarly divided between such labels and artworks (Smith et al., 2017) suggesting that the textual information is an important part of artwork experience. Also, the type of labels seems to be important. Labels focusing on the aesthetic context (aesthetic and interpretive interest) promote a more distributed gaze than standard labels providing simple background information (Bailey-Ross et al., 2019). Contextual detailed labels are associated with more switches between images and text and less time spent viewing artworks as opposed to factual labels or no labels at all (Lin and Yao, 2018). Providing labels rich in information disrupts focused attention on the paintings, but at the same time helps viewers to process them more easily. The placement of labels is another important factor. Reading labels on the walls is associated with longer viewing time for artworks as compared to reading labels on mobile devices, but the mobile labels allow a quicker review of the information (Yi et al., 2021). Finally, labels direct gaze to important parts of images, and their effect is stronger in the second phase of viewing, i.e., after the initial 2 s (Walker et al., 2017). This top-down guidance may be particularly important when images lack semantic cues (Davies et al., 2017) and it is connected with shorter fixation durations for artworks equipped with labels than artworks alone (Keller et al., 2020). Taken together, these results suggest that labels facilitate art perception by providing contextual cues on where and how to look at artworks.

The other source of contextual information are artworks’ titles. Despite the profusion of studies investigating the effects of titles on aesthetic judgments, there are surprisingly few studies that explored the effects of titles only on eye movements. In one of the first studies Franklin and colleagues found that the presence of titles did not influence where the participants reported they were looking (Franklin et al., 1993). However, results of more recent studies that employed modern eye-tracking suggest that titles guided eye movements to informative regions related to the titles (Hristova et al., 2011; Bubić et al., 2017). Bubić et al. (2017) saw that the presence of titles directs gaze to areas that are described by the titles (e.g., cannons within Kandinsky’s work *Improvisation 30, Cannons*). In particular, they noted that viewers familiar with titles focused on the informative areas of the paintings earlier (1.5 s vs. 2.4 s), spent more time looking at these areas and returned to them more frequently than viewers unfamiliar with the titles (Bubić et al., 2017). The authors also note that the title seems to play a role later on in the viewing, possibly during the second stage of processing (survey phase) that starts after the initial 2 s of viewing that are mostly devoted to analysis of structural and semantic properties (gist phase; Locher, 1996; Nodine and Krupinski, 2003; Locher et al., 2007). This late effect of titles is in accordance with most models of aesthetic processing as they all emphasize the fact that initially gaze is guided by bottom-up image properties and only later on the sense-making and cognitive mastering related to the top-down effect of titles takes place (for a review, see Pelowski et al., 2016). The importance of titles was confirmed also by Hristova et al. (2011) who used works by Dali and Caravaggio. They found that participants looked more at areas associated with titles (e.g., elephants in Dali’s *Swan reflecting elephants*), and the effect of titles was stronger for Dali’s works than for the works by Caravaggio. The authors attributed the more pronounced effect of titles in the case of Surrealist paintings to the higher degree of ambiguity that these works contain. In such a case the guidance provided by titles may be needed more than when the paintings contain little ambiguity.

The results of both of these studies are interesting, however, they don’t explain how the presence of titles influences other eye movement parameters, and they cannot address the dynamics between image and title because the titles were always presented beforehand. In this respect there is one study which offers more insight (Kapoula et al., 2009). The authors used three different conditions: untitled, active (participants had to invent a title) and driven (original title) and three paintings by Léger. They showed that original titles were associated with an increase in fixation durations for all paintings. This effect is interpreted in terms of increased cognitive activity because the original title encouraged participants to match the title to the painting. Moreover, saccade amplitude was bigger for one painting (*The Wedding*, Léger) which again is interpreted in terms of cognitive effort related to fitting the title to the painting. The fact that this particular painting produced a different type of results than other paintings is explained in terms of high visual (many elements) and semantic complexity (many meaningful recognizable objects dispersed in different parts of the canvas) that characterizes this painting. Therefore, it seems that the presence of titles should lead to longer fixations and bigger saccade amplitudes especially in the case of complex paintings. Finally, as in previous studies, it was found that titles guide the gaze to the informative regions of paintings (e.g., clock in *The Alarm Clock*, Léger).

Summing up, the literature on the effects of textual information on eye movement in art perception provide quite varied and sometimes inconsistent results. For example, some studies report that text accompanying images leads to longer fixations (Kapoula et al., 2009), whilst others report an opposite effect (Keller et al., 2020) or no effect at all (Davies et al., 2017). Researchers usually describe only a few eye movement parameters making comparison of results difficult and focus mostly on whether the titles guide gaze to informative regions, neglecting other aspects of eye movements. Finally, in most of the studies labels or titles are presented on a separate slide before the image is presented which impedes exploration of the dynamics between text and image. We propose that it would be useful to explore the relation between titles and paintings by placing titles underneath images. Such a setting not only resembles viewing art in a museum more but also allows us to trace the gaze dynamics between verbal and pictorial information.

Also, as titles influence aesthetic judgments in terms of understanding and liking (e.g., Russell, 2003; Swami, 2013; Bubić et al., 2017) it would be interesting to explore the relationship between eye movements and such judgments. Without trying to determine the direction of this relationship, we can assume that there is a reciprocal dynamics between where and how one looks and what one feels or thinks. Studies in empirical aesthetics have shown a quite complex and inconsistent image in this respect. For example, some authors report positive correlations between total fixation time, liking and interest (Mitschke et al., 2017), and between fixation durations and liking (Plumhoff and Schirillo, 2009). Other authors show no relationship between eye movement and aesthetic judgements (Heidenreich and Turano, 2010; Massaro et al., 2012; Gartus et al., 2015; Pelowski et al., 2018).

Brieber et al. (2014) demonstrated that longer viewing time for artworks and their labels is related not only to higher appreciation (combined scores of liking and interest), but also to greater understanding. Importantly, this relationship is particularly strong in a laboratory setting. Also, they found that artworks’ ambiguity predicted viewing time differently depending on the context: ambiguity was a positive predictor of viewing times in museums, but a negative one in a laboratory setting (Brieber et al., 2014). However, in a different study (in a laboratory setting) a positive relationship between subjectively experienced cognitive challenge (compound measure of ambiguity, complexity and inconsistency) and fixation durations, area of exploration and viewing time was found (Ganczarek et al., 2020). Therefore, longer careful fixations over large parts of paintings may be related to higher liking and greater understanding *via* a need to cognitively master artworks. Further interesting results are provided by Krejtz et al. (2014) who found that low dynamic entropy indicative of a predictable ordered way of viewing tends to be associated with higher appreciation of artworks and greater curiosity. Also, participants who appreciated certain artworks scanned paintings in a more balanced and uniform fashion evidenced by higher values of stationary entropy. Interestingly, Jankowski et al. (2020) obtained opposite results: in their study low stationary entropy (unbalanced attention dedicated to a few areas only) was related to greater appreciation.

The present study has three main aims. First, we aimed to investigate how the presence of titles affects eye movements. Usually researchers focus on how titles guide gaze to informative regions of paintings (Hristova et al., 2011; Bubić et al., 2017), but we were interested in exploring if the sole presence of titles changes eye movements. A similar approach was adopted by Kapoula et al. (2009) and to the best of our knowledge this is the only study that focused not only on where artwork titles guide gaze to, but also on how they affect eye movement parameters. Parameters such as fixation duration or saccades amplitudes and durations are sensitive to the level of processing and task difficulty (see Table 1), thus we can expect they will be affected by the presence of titles. However, the direction of this relationship is less clear cut. Some authors suggest that providing titles alleviates cognitive effort related to viewing artworks reducing fixation durations (Keller et al., 2020), whereas others state that adding titles leads to a deeper processing evidenced by longer fixations (Kapoula et al., 2009). Therefore, the average fixation durations may be longer or shorter in the titled condition compared to the untitled condition. Also, we can expect differences in saccades between titled and untitled conditions. In particular, Kapoula et al. (2009) found that saccade amplitudes were higher in the case of titled paintings than their untitled counterparts. Moreover, titles may be related to more switches within areas of images, possibly in order to track objects named in the titles (as in Hristova et al., 2011; Bubić et al., 2017). This may result in a less balanced attention (lower stationary entropy) and greater unpredictability of fixations (higher dynamic entropy). Such influence of titles may be particularly evident in the case of semantically violated images where titles are an important cue for interpretation (Hristova et al., 2011). It regards especially consistent titles that name objects appearing in paintings. Inconsistent titles, i.e., titles nominating objects different from those depicted in paintings wouldn’t provide such guidance.

**TABLE 1:** Relationship between selected eye movement parameters and cognitive processes.

Eye movement parameter	Related cognitive processes
Fixation duration	Usually, longer fixations are related to deeper, more effortful processing (Henderson, 2011), but in problem solving long fixations can be related to an impasse when no deeper processing is occurring (Knoblich et al., 2001). Also, successfully completed difficult task is associated with long fixations, but high workload and high stress is associated with shorter fixations (Holmqvist and Andersson, 2017). Longer fixations are also associated with pictorial medium identification which is a task requiring careful scanning (Sharvashidze and Schütz, 2020).
Saccade amplitude	Decreased amplitudes are related to more difficult tasks and effortful processing (May et al., 1990) as when viewers are asked to identify a particular artistic medium used in a painting (Sharvashidze and Schütz, 2020). However, larger saccade amplitudes can be related to presence of multiple meaningful visual cues (Goldberg et al., 2002).
Saccade duration	Increased saccade durations for more difficult tasks (e.g., blurred stimuli Vuori et al., 2004) and when the processing capacity is reduced (e.g., schizophrenia; Bestelmeyer et al., 2006).
Dynamic entropy	Measure of curiosity and appreciation - low dynamic entropy, i.e., less random and less frequent switches between AOIs are related to higher curiosity and greater appreciation of artworks (Krejtz et al., 2014).
Stationary entropy	Measure of interest and appreciation- high values of stationary entropy are related to an even distribution of attention between different AOIs and higher appreciation (Krejtz et al., 2014). However, Jankowski et al. (2020) found that stationary entropy negatively predicts appreciation of paintings.

Secondly, we aimed to explore the effects of ambiguities namely semantic violations and/or inconsistencies between titles and images on eye movements when viewing titled and untitled paintings (in the latter case only the effects of semantic violations can be studied). Both semantic violations and title inconsistencies increase the level of difficulty when viewing art. To the best of our knowledge this is the first eye-tracking study to assess the combined effect of such ambiguities on eye movements. Therefore, we can only speculate on the possible direction of the relationship between semantic violations/inconsistencies in titles and eye movements. Given that ambiguities make the processing more effortful (Ganczarek et al., 2020; Szubielska et al., 2021a) we could expect longer fixations, smaller saccade amplitudes and shorter durations. However, if we consider that semantic violations are related to multiple visual cues spread in different areas of paintings, we could also hypothesize that the saccade amplitudes will increase (Goldberg et al., 2002). Also, as such violations and title inconsistencies are not necessarily appreciated (Szubielska et al., 2021a), they may be related to higher dynamic entropy, but lower stationary entropy (Krejtz et al., 2014, but see Jankowski et al., 2020). Finally, semantic violations in images are known to affect eye movements early (Võ and Wolfe, 2013), whereas the influence of titles is usually exerted later on in viewing (Bubić et al., 2017; Walker et al., 2017). It is especially interesting if we consider that in some studies (Leder et al., 2006) titled paintings are shown for very brief periods of time (e.g., 1 s), raising doubts if such short exposure is sufficient to exert an effect on eye movements and aesthetic judgements. Here we are interested to see how early titles are fixated on and when semantic violations and inconsistencies in titles affect eye movements. In order to be able to trace this temporal dynamics we present titles together with paintings.

Thirdly, we aimed to investigate the relation between aesthetic judgements in terms of liking and understanding and eye movement parameters on paintings and titles (overall eye movement parameters) and paintings alone (entropy measures). The literature here is quite scarce and contradictory, but based on the research reviewed earlier we propose that liked paintings are scanned more thoroughly resulting in longer fixations (Ganczarek et al., 2020), low dynamic entropy and high stationary entropy (Krejtz et al., 2014). Such a viewing strategy would reflect a careful and predictable scanning with even distribution of attention between areas of interest. As understanding is positively correlated with liking (Szubielska et al., 2021a) we can expect a similar pattern of results for this measure. When it comes to the relation between liking/understanding and saccades we can assume that low understanding resulting from high difficulty (as in the case of semantic violations and inconsistencies in titles) will be related to increased saccade durations. The saccade amplitudes on the other hand, could either decrease in the case of not well understood paintings due to effortful processing or increase if we consider that such difficulty may stem from multiple visual cues spread in different areas both within paintings and in titles (see Table 1).

Summing up, we hypothesize that (H1) the presence of titles will affect eye movements (fixation duration, saccade amplitude, saccade duration, first fixation duration, low stationary, and high dynamic entropy), but the direction of this influence is not clear. (H1a) The effect of titles on eye movements will be particularly evident in the case of paintings with semantic violations accompanied by consistent titles acting as an important cue for interpretation; (H2) the presence of inconsistencies (semantic violations and/or inconsistent titles) will be related to a longer fixation durations, bigger saccade amplitudes and durations due to more effortful processing. Also, we propose that the presence of inconsistencies will influence stationary and dynamic entropy of fixations within images, (H3) the effect of titles and their inconsistencies will be most prominent after the initial 2 s of viewing, whereas the effect of semantic violations will be present earlier. Finally, we propose that high liking and understanding will be associated with longer fixation durations, higher saccade amplitude, and low dynamic entropy, but high stationary entropy (H4).

## Materials and Methods

### Participants

One hundred twenty-seven naïve participants (age range 18–53, 66 females, *M* = 26.96, *SD* = 7.76) with normal or corrected to normal vision took part in the experiment. They were recruited through adverts and rewarded with a bookshop voucher. Participants were either students (of 45 various majors) or graduates with no formal education in art. The research was approved by the Ethical Committee of the Pedagogical University of Krakow.

### Stimuli

**FIGURE 2:**
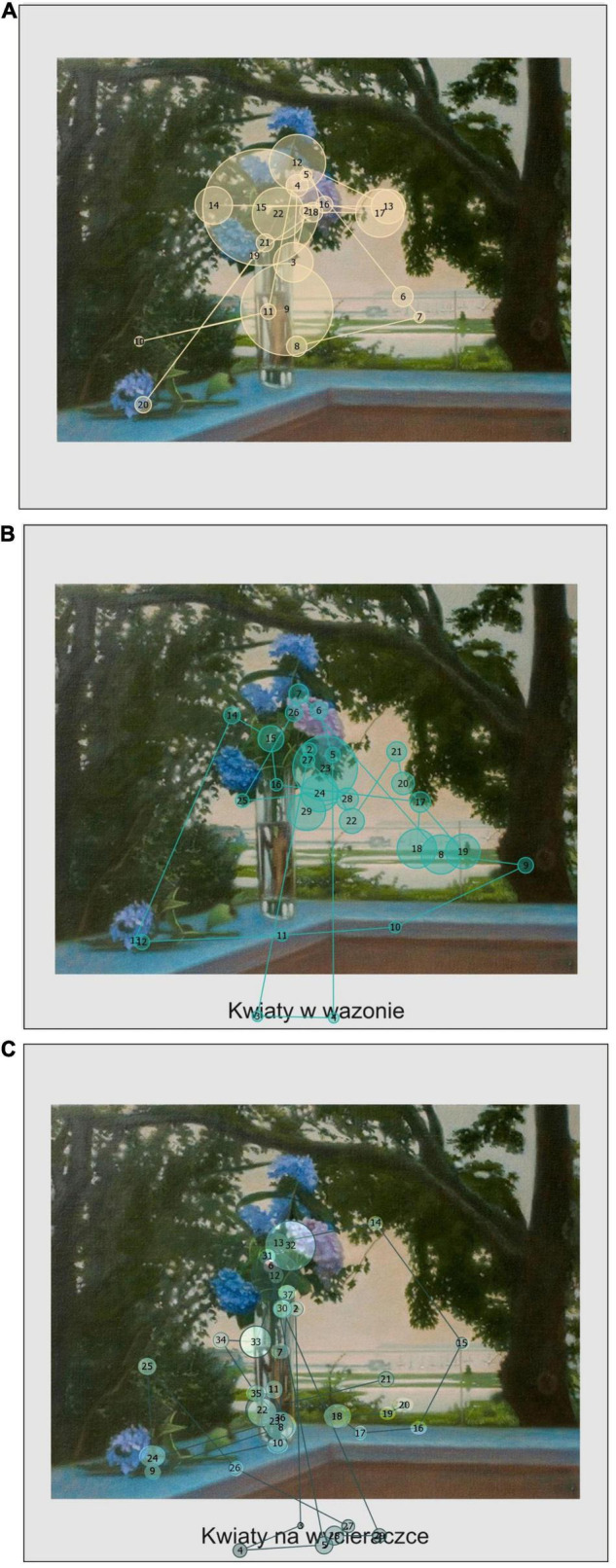
Examples of scanpaths from selected participants for a painting without semantic violations in three conditions. **(A)** Untitled, **(B)** consistent title (English translation: Flowers in a vase), **(C)** inconsistent title (English translation: Flowers on a doormat). The circles represent fixations, solid lines represent saccades, and the numbers correspond to indexes of fixations. The size of circles depicts relative fixation duration. Painting by Levenstein (2012), Orient at Dusk (Flowers in Glass Vase on Corner of Deck), oil on linen, 38 × 51 cm, courtesy of Matvey Levenstein and Galleria Lorcan O’Neill.

We used 40 digital reproductions of contemporary artworks. Twenty of them contained semantic violations and twenty did not. For instance, Jeff Koons’ *Niagara* (Koons, 2000; see Figure 1) is an example of a painting with semantic violations because it presented an unusual relationship between objects (e.g., plates with sweet pies and doughnuts placed next to women’s feet and a Niagara waterfall in the background). Instead, Matvey Levenstein’s *Orient at Dusk* (Levenstein, 2012; see Figure 2) is an example of an artwork without semantic violations because it is composed of typical objects in a scene (vase, flowers on a veranda) and there are no unusual relationships between objects. Images were chosen in a two-step procedure. First, we preselected 119 paintings which met the following criteria: were figurative, created after year 2000, displayed in leading art galleries or museums, and contained or not semantic violations (i.e., contained or not atypical objects with no reference to the global meaning of the scene or presenting unusual relationships between objects in the scene). Secondly, all the artworks were assessed by 8 independent experts in fine arts (art historians, visual artists). Judges rated from 1 (completely semantically inconsistent) to 7 (completely semantically consistent) paintings displayed in a random order in an online study. Subsequently, we choose the least semantically consistent images (*N* = 20, *M* = 2.08, *SD* = 1.26) and the most semantically consistent images (*N* = 20, *M* = 6.64, *SD* = 0.58). Kendall’s W coefficient was 0.87, suggesting high interjudge reliability. Each painting had three versions: untitled, consistent title, inconsistent title. Titles were also prepared in a two-step procedure. First, we produced six descriptive titles for each artwork, three consistent ones (naming elements presented in a painting, e.g., *Flowers in a vase*) and three inconsistent ones (naming elements absent in the painting, e.g., *Flowers on a doormat*). Afterwards 11 independent judges who were fine art experts (art historians and artists different than in the stimuli selection procedure) evaluated titles in an online study. After being instructed and given definition of consistent/inconsistent title (i.e., literally describes/does not describe what is presented in a painting) they used 1–7 Likert scale to rate each title. Eventually, two titles for each painting were selected: with the lowest score (inconsistent title *M* = 1.23, *SD* = 0.89) and the highest scores (consistent title *M* = 6.22, *SD* = 1.35). The interjudge reliability was high (Kendall’s W coefficient = 0.80). Full details of images (including original as well as fake consistent and inconsistent titles; Supplementary Table 1), and mean ratings by independent judges both for stimuli and title selection procedure are available in Supplementary Table 2. Please note that all images can be requested from the corresponding author.

Notably, the results of analyses of variance showed that the number of words and characters (we analyzed characters without spaces) in the titles did not differ significantly either depending on the type of title used (consistent vs. inconsistent) (both *p*s > 0.39), the type of painting (with vs. without semantic violations) (both *p*s > 0.09), or their interaction (both *p*s > 0.10). All the words used in the titles were known to participants (i.e., included in the National Corpus of Polish).

Finally, we checked if the two groups of images (with and without semantic violations) differed in terms of visual complexity. For this reason, we calculated two measures: complexity and image entropy. The complexity coefficient was based on histograms of oriented gradients following the procedure described by Redies et al. (2012). Image entropy was calculated with the *entropy* function in MATLAB (Gonzales et al., 2003). Both these measures provide a good estimate of visual complexity. We found that the two groups of paintings (with and without semantic violations) did not differ in terms of complexity [*t*(38) = −0.50, *p* = 0.62) and image entropy [*t*(38) = −0.26, *p* = 0.80). Complexity and image entropy values for each painting are listed in Supplementary Table 3.

### Procedure

Participants were informed about the procedure and gave written consent. They were randomly assigned to one of the three experimental conditions. The first group viewed the paintings with coherent titles (*n* = 42, *M* = 24.98 years, *SD* = 6.01; 23 women). The second group viewed the paintings with incoherent titles (*n* = 42, *M* = 26.38 years, *SD* = 7.77; 21 women). The control group saw the paintings with no titles (*n* = 43, *M* = 29.47 years, *SD* = 8.72; 22 women). Each participant viewed a total of 40 paintings (20 with semantic violation and 20 without). Before the experiment had started participants’ visual acuity, contrast sensitivity (Freiburg Visual Acuity and Contrast Test; Bach, 1996), and color vision (Barbur et al., 1994) were tested. Afterward, the eye-tracking session was performed. Participants were seated in front of a computer screen and eye-tracker. They were instructed to minimize their body and head movement during the eye-tracking recording, as well as having a trial session (with additional stimuli) before the experimental session. The eye movements data were recorded with the SensoMotoric Instruments iViewX™Hi-Speed500/1,250 eye tracker (500 Hz). During the test, the images were presented on a 27″ LCD monitor with Full HD resolution of 1,920 × 1,080. Images subtended a visual angle of 15.66 (vertical) by 10.5–26 (horizontal). Prior to the training session, a 13-point calibration was performed followed by a validation. A second calibration was performed after the initial 20 images were presented. Each image was displayed for 10 s. Before each new stimulus, the participant focused their gaze on the center of the screen where the fixation cross was displayed for 1 s. At the end a final validation was run in order to check for eventual calibration inaccuracies. After the presentation of each painting, participants evaluated images on two separate 7- point Likert scales, i.e., understanding scale (1—“I definitely don’t understand” to 7—“I definitely understand”) and liking scale (1—“I definitely don’t like” to 7—“I definitely like”). This part was not time-limited. The recording was carried out with the same environmental conditions, including lighting and acoustic insulation, for all participants. After the eye-tracking session, participants responded if they had seen any of the images before the experiment in order to control for familiarity with artworks. Moreover, they filled in questionnaires assessing personality traits (presented in Szubielska et al., 2021a) and answered demographic questions. Each session lasted approximately 60 min.

### Eye Movement Data Preparation

**FIGURE 3:**
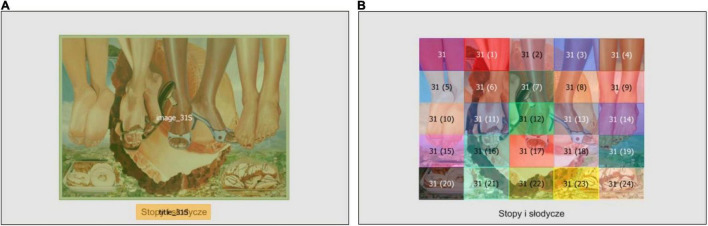
Examples of Areas of Interest (AOIs) used for the analyses of overall eye movement parameters, temporal dynamics of fixation, and the entropy measures; separate AOIs for image and title **(A)**, 5 × 5 gridded AOIs used for entropy measures **(B)**.

The eye movement data were sampled at 500 Hz with an average accuracy of 0.39 degree. Calibration accuracy was kept below 0.5 degree for all participants. Fixations and saccades were identified through a velocity-based detection algorithm, whilst blinks were removed. Eye movements were classified as saccades when they reached a peak velocity of 40 degrees/second. Fixations less than 80 ms were eliminated (as in Francuz et al., 2018; Jankowski et al., 2020) resulting in a loss of 1.4% of data. Moreover, additional calibration checks and offset correction were performed. Due to unsuccessful calibration 7.09% of data were removed. All analyses were run only on trials where participants viewed only paintings unknown to them (0.55% of data removed). Moreover, fixation durations exceeding 1,200 ms (0.6% of data), saccade amplitudes above 30 degrees (0.6%) and saccades durations above 100 ms (0.7% of data) were treated as outliers and removed from all analyses. After the initial data check, three types of areas of interest (AOIs) were created. For analyses of general eye movement parameters (for descriptive statistics see Table 2) and temporal dynamics of fixations each slide contained two AOIs: one for image and one for title (see Figure 3). AOIs around titles were drawn with a margin of 1.5 degree of visual angle in order to capture all fixations related to reading (Holmqvist and Andersson, 2017). Moreover, for analyses regarding the degree of entropy of fixations only images without text were used. Each image was divided in 5 × 5 rectangular AOIs resulting in 25 adjacent AOIs for a single image (see Figure 3; as in Jankowski et al., 2020). In order to address our aims, we performed three types of eye movement analyses. First, we analyzed summary data of fixations and saccades within images and titles (average fixation duration, saccade duration and amplitude, and first fixation duration analyzed for the untitled, consistent titles and inconsistent titles conditions), within titles only (average fixation on titles analyzed for the consistent titles and inconsistent titles conditions), and within images only (average fixation on images analyzed for three title conditions). Secondly, we analyzed raw data sampled at 500 Hz and detection of fixations on images or titles (analysis of time course of attention to titles analyzed only for the consistent title and inconsistent title conditions). Finally, we analyzed summary data of fixations within images (entropy of fixations within images analyzed for 3 title conditions).

**TABLE 2:** Descriptive statistics for the overall eye movement parameters grouped by semantic violations and title condition.

Eye movement parameter	*M* (*SD*)					
Condition	Semantic violations, untitled	No semantic violations, untitled	Semantic violations, consistent titles	No semantic violations, consistent titles	Semantic violations, inconsistent titles	No semantic violations, inconsistent titles
Average fixation duration (ms)	265.38 (140.48)	271.35 (150.05)	263.08 (147.22)	269.37 (155.58)	251.37 (140.49)	261.13 (152.69)
Average fixation duration on titles (ms)	–	–	174.16 (75.20)	175.37 (80.14)	175.34 (79.63)	180.37 (97.28)
Average fixation duration on images (ms)	265.53 (140.51)	271.41 (149.79)	276.31 (150.43)	283.10 (160.06)	264.67 (144.15)	274.12 (155.59)
First fixation duration (ms)	212.56 (113.72)	223.96 (124.85)	199.18 (107.84)	190.14 (92.20)	187.19 (106.12)	190.37 (110.61)
Saccade duration (ms)	42.97 (14.64)	42.95 (14.73)	44.30 (15.57)	44.78 (16.04)	44.78 (15.65)	45.20 (15.89)
Saccade amplitude (degrees)	4.00 (2.96)	4.16 (3.15)	4.47 (3.50)	4.67 (3.72)	4.60 (3.66)	4.74 (3.81)
Dynamic entropy	1.27 (0.22)	1.18 (0.23)	1.22 (0.22)	1.14 (0.24)	1.22 (0.23)	1.15 (0.24)
Stationary entropy	2.98 (0.50)	2.95 (0.61)	2.84 (0.52)	2.85 (0.57)	2.87 (0.51)	2.83 (0.59)

## Results

### Overall Eye Movement Parameters

Initially, in order to test the effects of titles and semantic violations on averaged standard eye movement measures (H1, H2, and H3) as well as the relationship between aesthetic judgements (liking, understanding) and eye movements (H4) the following eye movement parameters were computed: average fixation duration, average fixation duration on titles, average fixation duration on images, first fixation duration, saccade duration, and saccade amplitude. These measures were based on fixations and saccades to both images and titles except for the average fixation duration on titles and images which were computed from fixations landing only on titles and images, respectively. As first fixation duration we only used first fixations after the initial saccade. All initial saccades started in the middle of the screen. For this reason, 90.5% of the first fixations landed on images, and only 9.5% of them landed on titles. The descriptive statistics for all experimental conditions are presented in Table 2.

Separate mixed-effects linear models were run for each of the parameters with title condition (a categorical variable with three levels: untitled, consistent titles, and inconsistent titles), semantic violations (a categorical variable coded as absent and present), and their interaction as fixed effects. The model for the average fixation duration on titles was built only for the two titled conditions, i.e., consistent and inconsistent titles. As random effects we included participants, images, and random slopes for the effects of semantic violations within participants. All models were fitted with the *lmer* function from the lme4 R package (Bates et al., 2015). Initially, the best structure of random effects including random intercepts and slopes was evaluated by comparing models with and without selected random effects. Once the best random effects structure was chosen, models with and without the interaction between fixed factors were compared against each other. Most parsimonious models with lowest BIC values were selected for further analyses. Models’ comparisons were run with the *anova* function. We checked models’ assumptions and corrected problems with normality using a log transformation of the outcome variables.

We found that the overall average fixation duration (for full details, see Supplementary Table 4) was significantly predicted by the title condition [χ^2^(2) = 9.49, *p* = 0.01]: fixation durations were shortest in the inconsistent titles condition, followed by the consistent titles condition and the untitled condition. As can be seen in Figures 1, 2 when paintings were equipped with titles, the fixations were shorter than in the untitled condition. The only significant difference was between the untitled condition and the inconsistent titles condition (*b* = −0.07, *SE* = 0.02, *z* = −3.08, *p* = 0.01). Average fixation duration on titles was not significantly influenced by any of the predictors [χ^2^(1) = 0.11, *p* = 0.74 semantic violations and χ^2^(1) = 0.43, *p* = 0.51 titles condition]. Similarly, average fixation duration on images was not predicted by semantic violations [χ^2^(1) = 1.31, *p* = 0.25] or the title condition [χ^2^(2) = 1.29, *p* = 0.52]. Instead, first fixation duration was predicted by the title condition [χ^2^(2) = 24.26, *p* < 0.001]: the first fixation duration was significantly longer in the untitled condition than in both titled conditions (untitled vs. consistent titles *b* = 0.11, *SE* = 0.03, *z* = 3.21, *p* < 0.01, and untitled vs. inconsistent titles *b* = 0.17, *SE* = 0.04, *z* = 4.72, *p* < 0.001). The difference between two titled conditions was not significant (*b* = 0.05, *SE* = 0.04, *z* = 1.50, *p* = 0.29). Title condition also significantly predicted saccade durations [χ^2^(2) = 10.45, *p* < 0.01]: compared to the untitled condition saccades were longer in consistent titles condition (*b* = 0.03, *SE* = 0.01, *z* = 2.28, *p* = 0.059) and in the inconsistent titles condition (*b* = 0.04, *SE* = 0.01, *z* = 3.12, *p* < 0.01). No difference was found between two titled conditions (*b* = −0.01, *SE* = 0.01, *z* = −0.84, *p* = 0.68). Similarly, saccade amplitudes were higher (see Figures 1, 2) in both titled conditions as compared to no title condition [χ^2^(2) = 19.83, *p* < 0.001; untitled vs. consistent *b* = 0.10, *SE* = 0.03, *z* = 3.66, *p <* 0.001 and untitled vs. inconsistent *b* = 0.11, *SE* = 0.03, *z* = 4.01, *p* = < 0.001]. The difference between the two titled conditions was not significant (*b* = −0.01, *SE* = 0.03, *z* = −0.35, *p* = 0.94).

Finally, we checked if any of the overall eye movement measures was significantly predicted by two aesthetic judgments, i.e., liking and understanding. Liking and understanding were group mean centered. We found that neither of the parameters were significantly related to liking or understanding.

### Temporal Dynamics of Fixations

**FIGURE 4:**
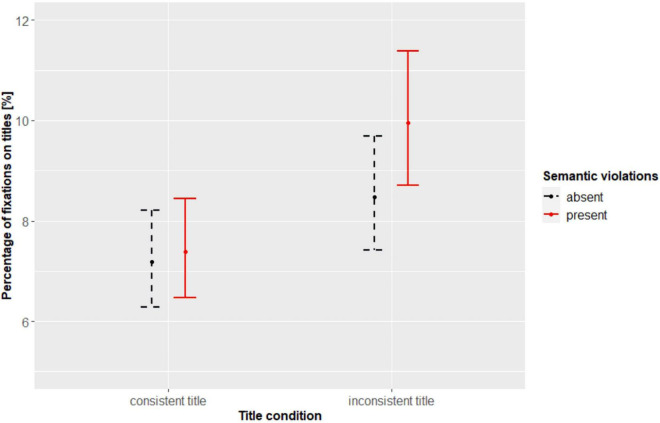
Percentage of fixations on titles depending on the presence of semantic violations and types of titles. The error bars represent 95% confidence intervals.

In order to test the temporal effects of titles and semantic violations (H3) a second type of analysis was run concerning the dynamics in time of eye movements distribution between images and titles. For this purpose, we used the *eyetrackingR* package (Dink and Ferguson, 2015) and analyzed data only from the titled conditions (i.e., consistent and inconsistent titles). We were interested to see if any differences related to the experimental conditions (semantic violations and types of titles) appeared during the course of viewing. Initially, analyses with data aggregated over time windows were run in order to see if the differences due to the experimental conditions were significant. In particular, we examined if the experimental manipulation affected the proportion of fixations to the titles and images. We found a significant interaction between semantic violations and title condition [χ^2^(1) = 8.86, *p* < 0.01; Figure 4]. Participants spent more time on titles when viewing semantically violated images with inconsistent titles than semantically non-violated images with both consistent titles (*b* = −0.32, *SE* = 0.09, *t* = −3.41, *p* < 0.01) and inconsistent titles (*b* = −0.16, *SE* = 0.03, *t* = −4.84, *p* < 0.001). Finally, titles were viewed more often in the semantically violated images with inconsistent titles than semantically violated with consistent titles (*b* = −0.30, *SE* = 0.09, *t* = −3.13, *p* = 0.01). As can be seen in Figure 1C the double inconsistency, i.e., semantic violations accompanied by an inconsistent title, are related to multiple fixations with later revisits. On the other hand, the consistent titles are rarely associated with revisits and usually fixated on only during the initial few seconds of viewing. Please note that the later revisits to titles occur only in a subgroup of participants.

Secondly, in order to see how early participants looked at titles depending on the condition we performed onset contingent analysis (e.g., Fernald et al., 2008). On average, people switched from image to a title at 936.28 ms (*SD* = 1060.41) and this switch time was similar irrespective of the presence of semantic violations [χ^2^(1) = 1.17, *p* = 0.28] or inconsistencies in titles [χ^2^(1) = 0.38, *p* = 0.54] given that both fixed effects were not significant.

**FIGURE 5:**
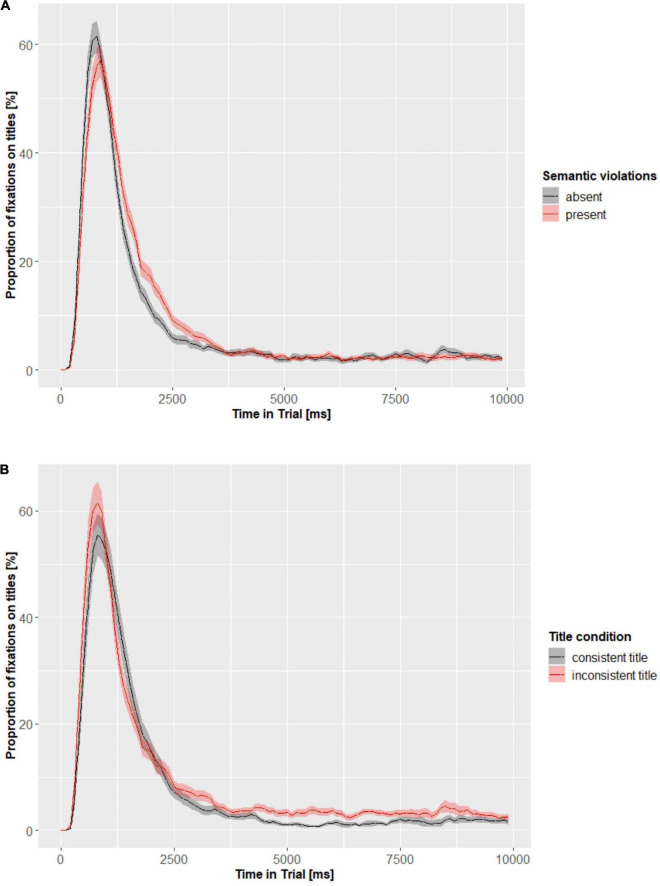
Divergence curves for the effects of semantic violations **(A)** and title condition **(B)** on the percentage of fixations on titles. The divergence curves illustrate time-course of the data summarized into time-bins (100 ms) with respect to percentage of fixations on titles depending on the experimental condition. The shading represents 95% confidence intervals.

Furthermore, in order to explore the course of attention to titles in respect to the experimental conditions we performed divergence analysis. Separate divergence curves for the effect of semantic violations (Figure 5A) and the title condition (Figure 5B) were computed. Figures 5A,B illustrate the course of painting viewing by all participants and thus indicate a typical viewing pattern. It can be seen that most fixations land on titles approximately 1,000 ms into the viewing and eventual later refixations are especially frequent for the inconsistent titles.

In order to assess the onset of a predictor’s effect on fixation to titles, a bootstrapped cluster-based permutation analysis was run (Maris and Oostenveld, 2007) with time bins of 100 ms and 1,000 bootstrapping samples. We found four potential clusters where the semantic violations could have an effect. Subsequent *t*-tests revealed, however, that only the first two clusters were significant. In particular, the differences emerged as early as 300–900 ms and later in the 1,200–2,800 ms time window. Images without semantic violations, compared to semantically violated images, were associated with more frequent glances to the titles between 300 and 900 ms (*p* < 0.01). Images with semantic violations instead received more frequent fixations on titles later between 1,200 and 2,800 ms (*p* < 0.001). In other words, semantic violations in images attracted attention early in the viewing thus directing attention to titles later on. In Figures 2B,C (painting without semantic violations) the 3rd fixation is already directed on the title. In the case of paintings with semantic violations (Figures 1B,C) the initial fixations on titles appear slightly later, i.e., around 4th fixation. Indeed, on average for paintings without semantic violations first fixation on titles was indexed as 3.31, compared to average index of 3.67 for first fixations on titles in the case of paintings with semantic violations. A fixation index corresponds to its position in a fixation sequence. Also, in Figure 5A the peak of the divergence curve for semantically violated paintings is shifted slightly to the right indicating that fixations on titles occurred here later than in the case of semantically non-violated paintings.

The cluster analysis for the types of titles revealed that there are potentially four clusters where the effect of inconsistencies in titles on fixations to titles could be in place. However, subsequent tests revealed that the effect of titles’ (in) consistency emerges in two time bins: 4,400–6,400 ms (*p* < 0.001) and 6,500–7,300 ms (*p* = 0.04). It suggests that from approximately 4–7 s into the viewing the inconsistent titles received more fixations than consistent titles. Therefore, compared to semantic violations, inconsistencies in titles attracted attention later on. This can be seen in Figures 1C, 2C where inconsistent titles are revisited at later stages of viewing (e.g., the inconsistent title in Figure 2C is revisited by the 27th fixation which corresponds approximately to 5 or 6 s into the viewing). Similarly, in Figure 5B the divergence curves show more frequent fixations on inconsistent than consistent titles.

### Dynamic and Stationary Entropy of Fixations

Finally, in order to investigate the effects of titles and semantic violations on the spatial distribution of fixations within paintings (H1, H1a, and H2) as well as in order to explore the relationship between aesthetic judgements (liking, understanding) and the spatial distribution of fixations (H4) we computed two measures as described by Krejtz et al. (2014). The first one was the coefficient related to stationary distribution of fixations (stationary entropy). This measure is indicative of the overall distribution of attention to different AOIs. Low values of stationary entropy correspond to a focused attention to few AOIs, whilst high values correspond to attention distributed more equally between different AOIs. The second measure was the dynamic entropy, i.e., a coefficient representing transitions between AOIs. This measure is modeled as a Markov chain describing individuals’ AOI switching patterns which can be predictable (low values) or unpredictable or random (high values). The entropy values were calculated for fixation distributions between 25 AOIs defined for each image by creating a 5 × 5 grid (see Figure 3). Two mixed effects models for the dynamic and stationary entropy were created with semantic violations (a categorical variable coded as absent and present) and title condition (a categorical variable with three levels: untitled, consistent titles, and inconsistent titles) as fixed effects. Only fixations on images were used. Moreover, as images had different widths (the height was kept constant between images), AOIs were of different sizes. In order to control for these differences, the image surface was added as a fixed effect to the models. Also, as entropy values increase with the number of fixations, we controlled the number of fixations by adding this variable to the model. As random effects we entered participants and paintings as well as random slopes for the effects of semantic violations within participants. The model selection procedure was identical to the one described for analysis of overall eye movement parameters. Table 2 shows descriptive statistics for the entropy of fixations measures.

The first model for dynamic entropy revealed a significant effect of semantic violations [χ^2^(1) = 5.42, *p* = 0.02] suggesting that images with semantic violations were related to more unpredictable fixation distribution. Moreover, title condition was a significant predictor of dynamic entropy [χ^2^(2) = 10.95, *p* < 0.01]: both consistent and inconsistent titles were related to more unpredictable fixations than the untitled condition (consistent vs. untitled *b* = 0.04, SE = 0.02, z = 2.68, *p* = 0.02, and inconsistent vs. untitled *b* = 0.045, SE = 0.02, z = 2.86, *p* = 0.01). The differences between two titled conditions were not significant (*b* = −0.00, *SE* = 0.02, *z* = −0.17, *p* = 0.99). The second model for the stationary entropy revealed that neither semantic violations [χ^2^(1) = 0.03, *p* = 0.87] nor title condition [χ^2^(2) = 0.46, *p* = 0.79] were significant predictors. It suggests that the attention allocation was similar between different conditions.

**FIGURE 6:**
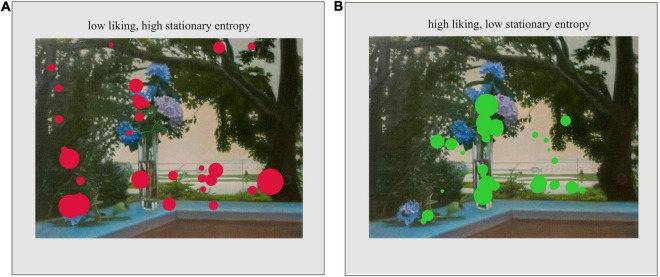
Examples of fixation distributions from selected participants for a disliked image with high stationary entropy of fixations **(A)**, and a liked image with low stationary entropy of fixations **(B)**. The circles represent fixations and their size represents relative fixation duration.

Finally, two separate models for ratings of liking and understanding with dynamic and stationary entropy as predictors were created. The models also included the number of fixations as a fixed effect, participant and images as random effects, and random slopes for the effects of dynamic and stationary entropy within participants. We found that liking was negatively predicted by the stationary entropy [χ^2^(1) = 5.55, *p* = 0.02], but not by the dynamic entropy [χ^2^(1) = 0.97, *p* = 0.32]. It suggests that an unbalanced attention (attention dedicated to specific AOIs) was related to higher liking. Moreover, the number of fixations was a positive predictor of liking [χ^2^(1) = 5.76, *p* = 0.02] indicating that fixations close to each other, possibly in a small amount of the most attractive AOIs, were related to a higher appreciation. Figure 6 shows a typical fixation distribution for a disliked (Figure 6A) and liked image (Figure 6B). In the case of liked images fixations are concentered on few attractive objects, whereas disliked images are associated with fixations spread in different areas.

The model for understanding demonstrated that neither type of entropy predicted the understanding [dynamic entropy χ^2^(1) = 2.26, *p* = 0.13 and stationary entropy χ^2^(1) = 0.06, *p* = 0.81].

## Discussion

Our results on the effects of titles and semantic violations on eye movements suggest a complex and interesting pattern. First of all, we found that the presence of titles, indeed, affects *how* paintings are viewed (H1) and not only *where* people look, which was already explored by other authors (Hristova et al., 2011; Bubić et al., 2017). In particular, the differences between titled and untitled conditions appear in the first fixation duration (shorter in the titled than the untitled conditions), average fixation duration (shorter fixations for the inconsistent titles than untitled condition) saccade durations and amplitudes (both bigger in the titled than in the untitled condition). Moreover, the titled conditions were associated with a greater degree of unpredictability of fixations (dynamic entropy) than the untitled condition. We also found that titles are fixated on average at 935.87 ms into the viewing which suggests that titles are an important part of artworks and that viewers look for additional cues early. Our results therefore confirm that the sole presence of text accompanying art affects the way people view it. Larger and longer saccades coupled with more unpredictable fixations follow the results by Lin and Yao (2018) who found that labels disrupt attention by favoring switches between images and text. Long saccades are also related to tracking multiple meaningful objects spread over different parts of images (Goldberg et al., 2002). In the case of paintings used in our study titles named objects contained in images and it is possible that presenting titles favored longer saccades driven by such objects fanned out in different areas of paintings. Also, shorter first fixations in the titled condition suggest that viewers would initially dwell less on images and move quickly to titles in search of interpretative cues. Instead, the short first fixation durations in the case of titles and shortest first fixations in the case of inconsistent titles are in accordance with the study by Keller et al. (2020), but contradicts findings by others (Kapoula et al., 2009). This discrepancy may be explained by differences in the types of titles used and the type of stimuli. Whilst Kapoula et al. (2009) used original titles that were both descriptive and metaphorical, we used fabricated ones that were descriptive in nature. Also, in their study only 3 abstract works were used, whilst both the study by Keller et al. (2020) and ours employed figurative paintings. It is possible that both differences in type of artworks and type of titles could account for the differences. When abstract art is accompanied by descriptive and metaphorical titles, the cognitive load may rise because viewers try to recognize objects and grasp a meaning. Thus, with increasing cognitive load fixation durations increase as well. Instead, in the case of figurative paintings object identification is not so challenging hence viewers may experience less effort when equipped with descriptive titles. In our study the inconsistent titles were associated with shortest fixations. These titles named objects that were not present in paintings which resulted in lower understanding and less appreciation (Szubielska et al., 2021a). Such a situation may be stressful because it interrupts viewers’ expectations. As a consequence, it could lead to short fixations that are typical for highly stressful difficult tasks (Holmqvist and Andersson, 2017). Moreover, we did not find significant differences between the untitled condition and the consistent titles condition in terms of fixation durations. It suggests that the short fixations related to inconsistent titles may be associated indeed with confusion and low understanding. This interpretation could be corroborated by the fact that in the study by Szubielska et al. (2021a) untitled and consistent titles condition did not differ in terms of understanding, only the inconsistent titles reduced understanding. Also, Keller et al. (2020) found shorter fixations for a condition in which an informative label with a metaphorical title accompanied a painting compared to viewing the painting alone. The authors propose that shorter fixations are related to easier processing because viewers were equipped with contextual information. In the case of inconsistent titles in our study it is possible they were treated as metaphors that could alleviate the cognitive effort. However, as inconsistent titles reduced understanding we could speculate that rather than reducing cognitive load, they increased it. The shorter fixations may be caused by more frequent refixations on titles and more chaotic scanning patterns which was evidenced by a higher degree of dynamic entropy.

The role of titles in the case of more difficult images (with semantic violations) (H1a) was partially confirmed. We found that titles (both consistent and inconsistent) of semantically violated paintings were fixated more often, but only in a particular moment of viewing (between 1,200 and 2,800 ms). The importance of titles for paintings that either lack semantic cues (Kapoula et al., 2009; Davies et al., 2017) or contain semantic violations (Hristova et al., 2011) has been documented, but the authors reported only effects over the whole period of viewing. In our study, over all time windows, most fixations landed only on inconsistent titles accompanying paintings with semantic violations, but the consistent titles for semantically violated paintings received as many fixations as titles for paintings without semantic violations. This result suggests that the presence of semantic violations does not equally (over the whole time of viewing) increase the need for contextual cues in terms of titles. Instead, the gaze is directed to titles mostly after the initial second of viewing. Most probably it is caused by the fact that first viewers had to notice the semantic violations and only subsequently could they look for additional cues. It is in line with the third hypothesis stating that the effects of semantic violations preceded those of inconsistencies in titles (H3). In fact, we saw that whilst semantic violations affected eye movements as early as 300 ms, inconsistencies in titles, on average, exert their influence after 4 s into the viewing. It means that the semantic violations in the images were detected early, but inconsistencies in the titles much later. This result follows the time course of art perception as proposed by the 2-stage processing model which differentiates between the mostly image-driven gist phase and more top-down survey phase (Locher, 1996; Nodine and Krupinski, 2003; Locher et al., 2007). The early effect of semantic violations mirrors the N300 and N400 components found by Võ and Wolfe (2013) in their study on the effects of semantic violations in real-life scenes. Our results confirm that the semantic violations are detected around 300 ms and this early effect lasts until 900 ms. This also encompasses the late effect (P600) found by Markey et al. (2019). We can speculate that in the case of our study, the semantic violations held viewers’ attention until the first fixations on titles which for semantically violated images appear after the initial second of viewing. Inconsistent titles on the other hand received most fixations later on in the viewing, when semantic violations were detected and semantic matching with titles could have been executed. Therefore, whilst titles in general are fixated quite early (just before 1 s into the viewing), a more in-depth processing of titles allowing to evaluate their consistency or inconsistency with a painting takes place later that is after 4 s. Our results are also in line with the model by Leder et al. (2004) and Leder and Nadal (2014) who propose that the initial stage of processing relies more on image properties and only subsequently is followed by a more elaborated cognitive processing stage where top-down influences take place. The later effect of titles was found by other authors (Bubić et al., 2017; Walker et al., 2017) who indicate that titles as top-down cues exert more influence after the initial 2 s of viewing. Our findings corroborate this late effect and also suggest that there is an interaction between type of titles and images. For paintings without semantic violations titles are fixated earlier and guide gaze to a lesser degree. Instead, semantic violations in paintings delay the influence of titles, because the violations themselves attract attention very early. But the same violations make the titles even more important in the second stage of viewing.

Furthermore, we found that inconsistent titles accompanying semantically violated images received most attention confirming our assumption that inconsistencies will attract attention (H2). We can speculate that the inconsistent titles were fixated in order either to search for meaningful cues to appreciate and comprehend an image or in order to resolve the ambiguity stemming from the incoherency between image and text. The consistent titles on the other hand did not provide any additional cues because they described objects appearing in images. Thus, there was no need to return to such a title. The inconsistent titles instead could have been used also as a metaphor for a meaning not readily available as in the case of semantically violated paintings. Interestingly, the images with double inconsistencies (titles and semantic violations) were associated with most switches between image and text which suggests that a very high degree of ambiguity provokes dispersed and chaotic viewing patterns.

The other type of inconsistencies, i.e., semantic violations, contrary to our predictions, were not related to increased fixation durations or bigger saccade amplitudes and durations (H2). It is interesting if we consider that the semantically violated images are perceived as very challenging (Ganczarek et al., 2020; Szubielska et al., 2021a), thus we should expect longer fixation durations (Henderson, 2007; Võ and Henderson, 2009; Ganczarek et al., 2020). We can speculate that it was the presence of titles that influenced the viewing patterns and rather than promoting a focused prolonged look to ambiguous areas of images it favored multiple switches between image and text. This interpretation is supported by the fact that the presence of titles was related to high dynamic entropy, characterized by unpredictable shifts of attention. In fact, in a previous study where paintings with semantic violations were presented without titles (Ganczarek et al., 2020), semantic violations alone did not significantly affect eye movements. Therefore, the presence of textual cues may have emphasized the semantic processing. However, rather than dwelling on semantically violated paintings, viewers used the titles to guide their perception characterized by multiple switches between text and images and short fixations. It suggests that text is a crucial factor that shapes how artworks are viewed and experienced. This interpretation is supported by the finding that dynamic entropy (unpredictability of fixations) was high for semantic violations. It means that the presence of semantic violations was disruptive and caused a more random exploration than in the case of paintings without semantic violations which possibly were explored in a more ordered way. It follows the findings by Võ and Henderson (2011) who noticed that semantic violations hinder search performance and make the eye movements less efficient in detecting targets. Our results suggest that the semantic violations provoke chaotic scanning patterns with multiple short fixations. It is possible that this particular way of viewing relates to a search for meaning which is difficult to obtain, particularly when semantic violations are coupled with inconsistent titles or have no titles at all.

Finally, our results on the relationship between aesthetic judgements (liking and understanding) and eye movements (H4) yielded an interesting picture. We found that liking was related to a lower degree of stationary entropy and higher number of fixations. This suggests that liking was associated with a more focused attention possibly directed to few adjacent areas of interest.

The significant relationship between liking and eye movements is in line with other studies (Plumhoff and Schirillo, 2009; Brieber et al., 2014; Mitschke et al., 2017). In general, a greater appreciation of artworks is related to attentive scanning as expressed by longer total fixation times (Mitschke et al., 2017), longer fixation durations (Plumhoff and Schirillo, 2009) and longer viewing times (Brieber et al., 2014). Our results confirm this and demonstrate that a greater liking is linked to careful scanning with a relatively narrow focus on few attractive areas. Interestingly, however, our results contradict results by Krejtz et al. (2014) who found that aesthetic appreciation is related to high values of stationary entropy reflecting an even distribution of attention between different areas of interest. Also, we did not replicate the negative relationship between dynamic entropy and liking. The differences may stem from employment of different and few stimuli (Krejtz et al., 2014 used one Impressionistic, one Renaissance and one Bauhaus painting) and different exposition time (30 s). It is possible that the composition of the three paintings used in their study influenced entropy values: in fact, the authors report that there was a significant effect of stimulus type on the entropy measures. Also, it is possible that longer viewing times result in different entropy values. The 10 s viewing time used in our study encompasses both the gist and survey phases of viewing, but it does not cover the subsequent free viewing characterized by greater scrutiny. We can speculate that with shorter viewing times liking is related to attention to few, attractive areas rather than a more distributed attention that may be present during long viewing times only for those who actually appreciate a given painting. Finally, our results are in accordance with the findings by Jankowski et al. (2020) who established that aesthetic appreciation is related to attention to a few specific areas of interest (low stationary entropy) and that there is no relationship between predictability of fixations (dynamic entropy) and appreciation. Therefore, our results suggest that liking may be associated with an uneven distribution of attention, guided by a few attractive areas rather than attention spread evenly as postulated by Krejtz et al. (2014). However, more research is needed in order to verify this claim.

Understanding on the other hand was not related to any eye movement parameters. In other studies, understanding and ambiguity were connected with longer viewing times (Brieber et al., 2014), whilst cognitive challenge (combined measure of image complexity, ambiguity and conflict) was associated with longer fixations and larger exploration area (Ganczarek et al., 2020). Our results suggest no relationship between the subjective feeling of understanding a painting and eye movements. This is despite the fact that semantic violations and inconsistent titles are rated as challenging and difficult to understand (Szubielska et al., 2021a).

Summing up, our results show that titles are an important part of paintings and that presenting artworks alone deprives viewers of the possibility to fully engage with artworks. Paraphrasing Pekarik (2004) we could say that: “because they had come for information, they took advantage of it.” Text provides a rich semantic context that allows an in-depth processing of both visual and verbal information. The combination of these two sources of information contributes to art perception, but at different stages. During the initial second of viewing semantic violations guided gaze most, after which the titles become useful tips for interpretation and dealing with inconsistencies. The role of titles seems to be most prominent especially when paintings contain semantic violations which are common in art and maybe contemporary art in particular.

Our study also has some limitations. First of all, we only used fake titles created on purpose for the study. Some participants in our study reported being suspicious about the titles which may have led to ignoring them, albeit the percentage of non-fixated titles was very low (0.21%). For future studies it would be interesting to compare the effects of inconsistent and consistent titles with original titles. Indeed, it is possible that the original titles may be treated differently by the viewers. Original titles are sometimes descriptive (e.g., *Tulips* by Alex Katz), but can also be inconsistent or metaphorical (e.g., *Brecht Play* by Csaba Nemes). Finally, original titles often contain the paradoxical *Untitled* title which is one of the most frequent titles in contemporary art and is used in various contexts such as showing non-reference or indicating autonomy of an artwork, to name a few examples (Vogt, 2006). It is possible that all these variations of original titles could affect eye movements differently. Also, titles are often considered not to be an addition, but a core element of an artwork (Levinson, 1985), hence presenting paintings without original titles limits our results. One could argue that in such a case the results inform about reactions to paintings and not artworks as a whole. On the other hand, sometimes paintings do not necessitate a title and are self-explanatory. In future studies it would be interesting to compare reactions to artworks where titles are deemed necessary, and artworks where titles are redundant. Secondly, our titles were descriptive, i.e., named objects appearing in the paintings. They could have reduced ambiguity in some cases by, for example, directing attention to a particular object and not others. However, in other cases, it could have been superfluous or, especially in the case of inconsistent titles, used as metaphors rather than literal descriptions. For this reason, it would be advantageous to investigate the role of metaphorical titles in perception of semantically violated paintings. We can speculate that in this instance the eye movements will be different than in the case of descriptive titles. Thirdly, in the current study we presented only titles, but it would be beneficial to include more elaborated labels with other details that can guide visual attention and cognitive mastering. Furthermore, our results pertain only to naïve viewers, whilst the titles or labels can have a different meaning for expert viewers. It is possible that for them this type of contextual information would be less important which has been noted in other studies (e.g., Szubielska and Sztorc, 2019). The heterogeneity of paintings used in our study was controlled in the random effects structure, but it would be interesting to create modified versions of artworks by purposely adding or removing semantic violations. This would add robustness to the experimental manipulation by keeping important variables such as size, color or contrast constant between conditions. Indeed, visual complexity influences the scanning behavior (e.g., Chassy et al., 2015) and including such a variable could provide insight into the relationship between visual and semantic complexity. Also, the images differed in terms of content. In future studies it would be advantageous to test the effects of specific content (e.g., human, landscapes, still life) on eye movements. In this respect it may be very interesting to include measures of individual differences, as they seem to influence what type of objects people focus their gaze on (De Haas et al., 2019). One could argue that the images also differ in respect to their artistic quality. This is an aspect that should be taken into account in future studies because it is possible that the aesthetic quality can influence not only aesthetic judgements (Pelowski et al., 2017), but also eye movements (Locher et al., 2015). Last, but not least our study focuses on semantic violations and inconsistency between image and title. Such inconsistencies are only a part of the ambiguity that contemporary art presents. Future studies should explore diverse strategies which artists apply to evoke ambiguity in their works.

## Data Availability Statement

The raw data supporting the conclusions of this article will be made available by the authors, without undue reservation.

## Ethics Statement

The studies involving human participants were reviewed and approved by Ethical Committee, Institute of Psychology, Pedagogical University of Cracow. The participants provided their written informed consent to participate in this study.

## Author Contributions

JG, KP, and MS contributed to conception and design of the study. KP, JG, and AS collected the data. JG and AS prepared the database. JG supervised the preparation of the manuscript. JG, KP, AS, and MS wrote sections of the manuscript. All authors contributed to manuscript revision, read, and approved the submitted version.

## Conflict of Interest

The authors declare that the research was conducted in the absence of any commercial or financial relationships that could be construed as a potential conflict of interest.

## Publisher’s Note

All claims expressed in this article are solely those of the authors and do not necessarily represent those of their affiliated organizations, or those of the publisher, the editors and the reviewers. Any product that may be evaluated in this article, or claim that may be made by its manufacturer, is not guaranteed or endorsed by the publisher.
