# Supplementary material for: Titles and Semantic Violations Affect Eye Movements When Viewing Contemporary Paintings
Source: Front Hum Neurosci. 2022 Mar 4;16:808330. doi: 10.3389/fnhum.2022.808330 (PMC8930854; doi:10.3389/fnhum.2022.808330)
Supplement: Supplementary file 1 [file Data_Sheet_1.docx]

# Supplementary materials

Digital copies of artworks can be requested from the corresponding author.

# Supplementary Table 1 List of images and titles used in the study

| Paintings without semantic violations | Title coherent with painting | Title incoherent with painting | Title coherent with painting (in Polish, as used in the study) | Title incoherent with painting (in Polish, as used in the study) |
| --- | --- | --- | --- | --- |
| Andersson Mamma, Karin 2014, *The one* | Swans on the water | Birds in a desert | Łabędzie na wodzie | Ptaki na pustyni |
| Blamowska, Małgorzata 2016, *Untitled* | Party participants | Women with a scooter | Uczestnicy przyjęcia | Kobieta z hulajnogą |
| Bocek, Anna 2015, *On the way to HK* | Man on a blue background | Woman on a blue background | Mężczyzna na niebieskim tle | Kobieta na niebieskim tle |
| Cafagna, Lucianella 2011, *Allons Enfant* | Child looking into the distance | Child facing forward | Patrzące w dal dziecko | Dziecko przodem |
| Currin, John 2001, *The gardeners* | A couple of gardeners | Couple of musicians | Para ogrodników | Para muzyków |
| Dao, Lee Chen 2006, *Lounge Bar* | Players in a bar | Pool players | Gracze w barze | Grający w bilard |
| Galieote, Danny 2012, *Hollywood Bound* | Relax on the sand | People on a carousel | Relaks na piasku | Ludzie na karuzeli |
| Havenkost, Eberhard 2012, *Mondsteine* | Grey stones | Pink stones | Szare głazy | Różowe głazy |
| Katz, Alex 2013, *Tulips* | Yellow tulips | Patch with cucumbers | Żółte tulipany | Grządka z ogórkami |
| Levenstein, Matvey 2012, *Orient at dusk* | Flowers in a vase | Flowers on a doormat | Kwiaty w wazonie | Kwiaty na wycieraczce |
| Materka, Bartek 2012, *Bez tytułu. Ona śpi [Untitled. She sleeps*] | Sleeping girl | Dancing on the couch | Śpiąca dziewczyna | Taniec na kanapie |
| Ozeri, Yigal 2010, *Untitled (Lizzie series)* | Long haired girl | Girl in a bath | Długowłosa dziewczyna | Dziewczyna w wannie |
| Popper, Simon 2008, *Fun guy Sketch n7* | Mushrooms in reds | Colony of frogs | Grzybki w czerwieni | Kolonia żab |
| Sasnal, Wilhelm 2006, *Untitled* | Table and a cigarette | Table and pasta | Stół i papieros | Stół i makaron |
| Schoeler, Christian 2009, *Untitled* | Young man | Dressed boy | Młody mężczyzna | Ubrany chłopak |
| Walker, Caroline 2010, *Conservation* | Reflection in a mirror | Figure in a park | Odbicie w lustrze | Postać w parku |
| Weaver, Grace 2015, *Sunday brunch* | Morning toilette | Figures in a kitchen | Poranna toaleta | Postacie w kuchni |
| Wood, Jonas 2012, *Interior with fireplace* | Interior with a fireplace | Spacious bathroom | Wnętrze z kominkiem | Przestronna łazeienka |
| Yiadom-Boakye, Lynette 2014, *The myriad motives of Man* | Dark skinned observers | Light skinned observers | Czarnoskórzy obserwatorzy | Jasnoskórzy obserwatorzy |
| Zhiying, Shi 2010, *Mars* | Stone landscape | Vegetal landscape | Kamienny krajobraz | Roślinny krajobraz |

| Paintings with semantic violations | Title coherent with painting | Title incoherent with painting | Title coherent with painting (in Polish, as used in the study) | Title incoherent with painting (in Polish, as used in the study) |
| --- | --- | --- | --- | --- |
| Brauning, Sascha 2009, *No solutions* | Section through a head | Section through a stomach | Przekrój przez głowę | Przekrój przez żołądek |
| Gagnon, Jacub 2009, *Storming the castle* | Roe deer and a castle | Objects of natural size | Sarna i zamek | Obiekty naturalnej wielkości |
| Gamarra, Sandra 2008, *Pilgrim* | In the museum | In the laundry | W muzeum | W pralni |
| Gay Jr, Serge 2005, *Pick of the liquor* | Girl in a bar | Girl behind the looms | Dziewczyna za barem | Dziewczyna za krosnami |
| Hawtin, Chris 2012, *Dredger* | Machine and a hut | Machine and a skyscraper | Maszyna i chatka | Maszyna i wieżowiec |
| Hearman, Louise 2003, *Untitled 986* | Strawberry over a street | Strawberry over a bathtub | Truskawka nad ulicą | Truskawka nad wanną |
| Hearman, Louise 2009, *Untitled 1280* | Cat over a wing | Plane and a dog | Kot nad skrzydłem | Samolot i pies |
| Humphrey, David 2002, *Kitties* | Bread slices and kitties | Camels and bread | Kromki i kociaki | Wielbłądy i chleb |
| Juszkiewicz, Ewa 2014, *Portrait of Carol Rama* | Plaits on the face | Plaits and tummy | Warkocze na twarzy | Warkocze i brzuch |
| Koons, Jeff 2000, *Niagara* | Feet and sweets | Feet and cauliflowers | Stopy i słodycze | Stopy i kalafiory |
| Krut, Ansel 2007, *Saucepan with spilled sausages* | Pot turned upside down | Sausages covering a pot | Odwrócony garnek | Serdelki przykrywające garnek |
| Martinez, Eddie 2012, *Time Assassin* | Man with a clock | Running kettle | Człowiek z zegarem | Biegnący czajnik |
| Nemes, Csaba 2013, Far from the Sea | Levitating in the city | Levitating on the beach | Lewitujący w mieście | Lewitujący na plaży |
| Nemes, Csaba 2014, Brecht Play | People and the street | Women with a car | Ludzie i ulica | Kobiety z samochodem |
| Popper, Simon 2008, *I'm not as green as i'm cabbage looking* | Cabbage instead of a head | Radish instead of a head | Kapusta zamiast głowy | Rzodkiewka zamiast głowy |
| Reeder, Scott 2011, *Bombs (at dinner)* | Bomb and dynamite | Objects on a bench | Bomba i dynamit | Przedmioty na ławce |
| Shimoda, Hikari 2012, *Children of this planet* | Multicoloured eyes | Brown eyes | Różnokolorowe oczy | Piwne oczy |
| Tansey, Mark 2011, *Invisible hand* | Mountain with a hand | Giant foot | Góra z dłonią | Gigantyczna stopa |
| Wood, Chris 2013, *Houdini* | Hypnotist in McDonalds | Contrabassist in McDonalds | Hipnotyzer w McDonalds | Kontrabasista w McDonalds |
| Viezens, Steve 2011, *Schmuckstück* | Car and bricks | Motorbike on a wall | Samochód i cegły | Motocykl na murze |

# Supplementary Table 2 Mean ratings by independent judges of semantic and titles (in)consistencies

| Paintings without semantic violations | Mean rating of semantic (in)consistencies (1 – semantically inconsistent to 7 – semantically consistent) | Mean rating of consistent titles (1 – inconsistent to 7 – consistent) | Mean rating of inconsistent titles |
| --- | --- | --- | --- |
| Andersson Mamma, Karin 2014, *The one* | 6.00 | 6.72 | 1.18 |
| Blamowska, Małgorzata 2016, *Untitled* | 6.71 | 6.09 | 1.00 |
| Bocek, Anna 2015, *On the way to HK* | 6.88 | 6.45 | 1.00 |
| Cafagna, Lucianella 2011, *Allons Enfant* | 6.86 | 6.54 | 1.64 |
| Currin, John 2001, *The gardeners* | 6.71 | 5.82 | 1.63 |
| Dao, Lee Chen 2006, *Lounge Bar* | 6.63 | 6.00 | 1.09 |
| Galieote, Danny 2012, *Hollywood Bound* | 6.66 | 6.27 | 1.00 |
| Havenkost, Eberhard 2012, *Mondsteine* | 5.43 | 6.54 | 1.18 |
| Katz, Alex 2013, *Tulips* | 6.13 | 6.73 | 1.00 |
| Levenstein, Matvey 2012, *Orient at dusk* | 6.71 | 6.27 | 1.09 |
| Materka, Bartek 2012, *Bez tytułu. Ona śpi [Untitled. She sleeps*] | 7.00 | 6.27 | 1.54 |
| Ozeri, Yigal 2010, *Untitled (Lizzie series)* | 7.00 | 6.72 | 1.00 |
| Popper, Simon 2008, *Fun guy Sketch n7* | 5.86 | 5.82 | 1.00 |
| Sasnal, Wilhelm 2006, *Untitled* | 6.58 | 6.54 | 1.18 |
| Schoeler, Christian 2009, *Untitled* | 6.86 | 6.18 | 1.00 |
| Walker, Caroline 2010, *Conservation* | 6.86 | 6.64 | 1.09 |
| Weaver, Grace 2015, *Sunday brunch* | 6.43 | 6.45 | 1.27 |
| Wood, Jonas 2012, *Interior with fireplace* | 6.83 | 6.72 | 1.00 |
| Yiadom-Boakye, Lynette 2014, *The myriad motives of Man* | 6.86 | 6.27 | 1.00 |
| Zhiying, Shi 2010, *Mars* | 6.50 | 6.45 | 1.27 |

| Paintings with semantic violations | Mean rating of semantic (in)consistencies (1 – semantically inconsistent to 7 – semantically consistent) | Mean rating of consistent titles (1 – inconsistent to 7 – consistent) | Mean rating of inconsistent titles |
| --- | --- | --- | --- |
| Brauning, Sascha 2009, *No solutions* | 1.86 | 6.00 | 1.64 |
| Gagnon, Jacub 2009, *Storming the castle* | 1.25 | 6.00 | 1.36 |
| Gamarra, Sandra 2008, *Pilgrim* | 2.43 | 6.09 | 1.09 |
| Gay Jr, Serge 2005, *Pick of the liquor* | 2.00 | 6.27 | 1.18 |
| Hawtin, Chris 2012, *Dredger* | 1.71 | 5.55 | 1.82 |
| Hearman, Louise 2003, *Untitled 986* | 3.00 | 6.27 | 1.27 |
| Hearman, Louise 2009, *Untitled 1280* | 1.71 | 6.09 | 1.45 |
| Humphrey, David 2002, *Kitties* | 1.86 | 6.27 | 1.18 |
| Juszkiewicz, Ewa 2014, *Portrait of Carol Rama* | 1.83 | 6.54 | 1.18 |
| Koons, Jeff 2000, *Niagara* | 1.34 | 6.45 | 1.09 |
| Krut, Ansel 2007, *Saucepan with spilled sausages* | 2.50 | 5.64 | 1.54 |
| Martinez, Eddie 2012, *Time Assassin* | 1.71 | 5.54 | 1.27 |
| Nemes, Csaba 2013, Far from the Sea | 2.25 | 6.45 | 1.00 |
| Nemes, Csaba 2014, Brecht Play | 2.43 | 5.82 | 1.00 |
| Popper, Simon 2008, *I'm not as green as i'm cabbage looking* | 1.75 | 6.36 | 1.18 |
| Reeder, Scott 2011, *Bombs (at dinner)* | 2.63 | 6.82 | 1.73 |
| Shimoda, Hikari 2012, *Children of this planet* | 2.43 | 5.91 | 1.36 |
| Tansey, Mark 2011, *Invisible hand* | 2.75 | 6.09 | 1.09 |
| Wood, Chris 2013, *Houdini* | 2.57 | 5.09 | 1.64 |
| Viezens, Steve 2011, *Schmuckstück* | 2.71 | 6.09 | 1.00 |

Supplementary Table 3 Visual complexity and entropy ratings for paintings

| Paintings without semantic violations | Complexity^1^ | Entropy^2^ |
| --- | --- | --- |
| Andersson Mamma, Karin 2014, *The one* | 0.72 | 7.00 |
| Blamowska, Małgorzata 2016, *Untitled* | 1.13 | 7.56 |
| Bocek, Anna 2015, *On the way to HK* | 1.18 | 6.96 |
| Cafagna, Lucianella 2011, *Allons Enfant* | 0.37 | 7.32 |
| Currin, John 2001, *The gardeners* | 0.97 | 7.34 |
| Dao, Lee Chen 2006, *Lounge Bar* | 1.16 | 6.61 |
| Galieote, Danny 2012, *Hollywood Bound* | 2.00 | 7.64 |
| Havenkost, Eberhard 2012, *Mondsteine* | 0.77 | 7.10 |
| Katz, Alex 2013, *Tulips* | 1.33 | 6.65 |
| Levenstein, Matvey 2012, *Orient at dusk* | 1.12 | 7.24 |
| Materka, Bartek 2012, *Bez tytułu. Ona śpi [Untitled. She sleeps*] | 1.27 | 6.89 |
| Ozeri, Yigal 2010, *Untitled (Lizzie series)* | 0.92 | 7.38 |
| Popper, Simon 2008, *Fun guy Sketch n7* | 0.51 | 4.34 |
| Sasnal, Wilhelm 2006, *Untitled* | 0.58 | 5.88 |
| Schoeler, Christian 2009, *Untitled* | 0.37 | 7.32 |
| Walker, Caroline 2010, *Conservation* | 0.86 | 7.42 |
| Weaver, Grace 2015, *Sunday brunch* | 0.57 | 6.49 |
| Wood, Jonas 2012, *Interior with fireplace* | 2.50 | 7.64 |
| Yiadom-Boakye, Lynette 2014, *The myriad motives of Man* | 0.39 | 5.91 |
| Zhiying, Shi 2010, *Mars* | 1.91 | 7.35 |

^1^ Complexity is calculated based on histograms of oriented gradients (Redies et al., 2012)

^2^ Entropy is calculated with the *entropy* function in MATLAB (Gonzales et al., 2003)

| Paintings with semantic violations | Complexity^1^ | Entropy^2^ |
| --- | --- | --- |
| Brauning, Sascha 2009, *No solutions* | 0.59 | 6.26 |
| Gagnon, Jacub 2009, *Storming the castle* | 0.56 | 3.18 |
| Gamarra, Sandra 2008, *Pilgrim* | 1.45 | 7.77 |
| Gay Jr, Serge 2005, *Pick of the liquor* | 2.25 | 7.60 |
| Hawtin, Chris 2012, *Dredger* | 2.09 | 7.09 |
| Hearman, Louise 2003, *Untitled 986* | 0.91 | 6.85 |
| Hearman, Louise 2009, *Untitled 1280* | 0.81 | 7.68 |
| Humphrey, David 2002, *Kitties* | 0.56 | 7.23 |
| Juszkiewicz, Ewa 2014, *Portrait of Carol Rama* | 0.69 | 6.58 |
| Koons, Jeff 2000, *Niagara* | 1.42 | 7.61 |
| Krut, Ansel 2007, *Saucepan with spilled sausages* | 0.67 | 6.40 |
| Martinez, Eddie 2012, *Time Assassin* | 2.26 | 7.74 |
| Nemes, Csaba 2013, Far from the Sea | 1.18 | 7.28 |
| Nemes, Csaba 2014, Brecht Play | 1.16 | 6.32 |
| Popper, Simon 2008, *I'm not as green as i'm cabbage looking* | 0.98 | 7.17 |
| Reeder, Scott 2011, *Bombs (at dinner)* | 0.66 | 7.02 |
| Shimoda, Hikari 2012, *Children of this planet* | 0.63 | 6.95 |
| Tansey, Mark 2011, *Invisible hand* | 1.00 | 7.52 |
| Wood, Chris 2013, *Houdini* | 1.89 | 7.73 |
| Viezens, Steve 2011, *Schmuckstück* | 1.36 | 7.60 |

^1^ Complexity is calculated based on histograms of oriented gradients (Redies et al., 2012)

^2^ Entropy is calculated with the *entropy* function in MATLAB (Gonzales et al., 2003)

Supplementary Table 4 Fixed effects from linear mixed-effects models

| Measure | Fixed effect | b | β | df | t | p |
| --- | --- | --- | --- | --- | --- | --- |
| Average fixation duration^1^ | Intercept | 5.51 | 0.78 | 157.65 | 278.51 | < .001 |
|  | Semantic violations^2^ | -0.02 | -0.02 | 39.23 | -1.39 | .15 |
|  | Consistent titles^3^ | -0.04 | -0.02 | 118.73 | -1.52 | .13 |
|  | Inconsistent titles^3^ | -0.07 | -0.05 | 118.44 | -3.08 | <.01 |
| Average fixation duration on titles^1^ | Intercept | 5.08 | 0.68 | 130.00 | 235.60 | < .001 |
|  | Semantic violations^2^ | -0.01 | -0.01 | 69.24 | -0.33 | .74 |
|  | Inconsistent titles^4^ | 0.02 | 0.02 | 99.55 | 0.66 | .51 |
|  | Semantic violations^2^ x Inconsistent titles^4^ | -0.00 | -0.00 | 9195.00 | -0.23 | .82 |
| Average fixation duration on images^1^ | Intercept | 5.51 | 0.77 | 144.66 | 253.77 | < .001 |
|  | Semantic violations^2^ | -0.02 | -0.02 | 37.97 | -1.15 | .26 |
|  | Consistent titles^3^ | 0.02 | 0.02 | 120.69 | 0.95 | .35 |
|  | Inconsistent titles^3^ | -0.00 | -0.00 | 120.35 | -0.08 | .93 |
| First fixation duration^1^ | Intercept | 5.29 | 0.74 | 121.53 | 167.48 | < .001 |
|  | Semantic violations^2^ | -0.01 | -0.01 | 38.40 | -0.26 | .80 |
|  | Consistent titles^3^ | -0.12 | -0.10 | 118.65 | -3.35 | < .001 |
|  | Inconsistent titles^3^ | -0.17 | -0.14 | 118.91 | -4.79 | < .001 |
| Saccade duration^1^ | Intercept | 3.71 | 0.76 | 120.00 | 276.82 | < .001 |
|  | Semantic violations^2^ | -0.01 | -0.01 | 39.76 | -0.49 | .63 |
|  | Consistent titles^3^ | 0.03 | 0.04 | 123.59 | 2.36 | .02 |
|  | Inconsistent titles^3^ | 0.04 | 0.05 | 123.83 | 3.18 | < .01 |
| Saccade amplitude | Intercept | 1.14 | 0.62 | 80.64 | 34.86 | < .001 |
|  | Semantic violations^2^ | -0.02 | -0.02 | 28.37 | -0.59 | .56 |
|  | Consistent titles^3^ | 0.10 | 0.06 | 122.54 | 3.85 | < .001 |
|  | Inconsistent titles^3^ | 0.11 | 0.07 | 122.51 | 4.16 | < .001 |
| Average fixation duration^1^ | Intercept | 5.47 | 0.74 | 141.90 | 437.54 | < .001 |
|  | Liking^5^ | -0.00 | 0.00 | 134300.00 | -0.04 | .97 |
|  | Intercept | 5.47 | 0.74 | 142.30 | 438.19 | < .001 |
|  | Understanding^5^ | 0.00 | 0.00 | 107000.00 | 1.44 | .15 |
| Average fixation duration on titles | Intercept | 5.08 | 0.68 | 102.90 | 359.06 | < .001 |
|  | Liking^5^ | -0.00 | -0.00 | 7975.00 | -1.33 | .18 |
|  | Intercept | 5.08 | 0.68 | 103.40 | 357.63 | < .001 |
|  | Understanding^5^ | 0.00 | 0.00 | 5755.00 | 0.58 | .56 |
| Average fixation duration on images | Intercept | 5.51 | 0.77 | 125.90 | 402.96 | < .001 |
|  | Liking^5^ | 0.00 | 0.00 | 119300.00 | 0.44 | .97 |
|  | Intercept | 5.51 | 0.77 | 126.10 | 403.37 | < .001 |
|  | Understanding^5^ | 0.00 | 0.00 | 104800.00 | 0.97 | .33 |
| First fixation duration | Intercept | 5.47 | 0.74 | 141.90 | 437.54 | < .001 |
|  | Liking^5^ | -0.00 | 0.00 | 134300.00 | -0.04 | .97 |
|  | Intercept | 5.47 | 0.74 | 142.30 | 438.19 | < .001 |
|  | Understanding^5^ | 0.00 | 0.00 | 107000.00 | 1.44 | .15 |
| Saccade duration^1^ | Intercept | 3.73 | 0.78 | 103.50 | 430.34 | < .001 |
|  | Liking^5^ | -0.00 | -0.00 | 128200.00 | -1.32 | .19 |
|  | Intercept | 3.73 | 0.78 | 103.10 | 429.45 | < .001 |
|  | Understanding^5^ | 0.00 | 0.00 | 111100.00 | 1.14 | .26 |
| Saccade amplitude^1^ | Intercept | 1.21 | 0.65 | 72.38 | 54.64 | < .001 |
|  | Liking^5^ | -0.00 | -0.00 | 130100.00 | -0.79 | .43 |
|  | Intercept | 1.21 | 0.65 | 72.17 | 54.52 | < .001 |
|  | Understanding^5^ | 0.00 | 0.00 | 123300.00 | 1.41 | .16 |
| Dynamic entropy | Intercept | 0.55 | -0.26 | 204.40 | 18.17 | < .001 |
|  | Semantic violations^2^ | 0.07 | 0.28 | 44.59 | 2.33 | .02 |
|  | Consistent titles^3^ | 0.04 | 0.18 | 125.00 | 2.40 | .02 |
|  | Inconsistent titles^3^ | 0.05 | 0.18 | 125.50 | 3.15 | < .01 |
|  | Image surface^5^ | -0.19 | -0.13 | 37.00 | -2.31 | .03 |
|  | Number of fixations | 0.02 | 0.52 | 2473.00 | 30.34 | < .001 |
|  | Semantic violations^2^ * consistent titles^3^ | -0.00 | -0.01 | 119.10 | -0.10 | .92 |
|  | Semantic violations^2^ * Inconsistent titles^3^ | -0.02 | -0.10 | 120.00 | -1.56 | .12 |
| Stationary entropy | Intercept | 1.97 | -0.02 | 101.60 | 23.25 | < .001 |
|  | Semantic violations^2^ | -0.02 | -0.03 | 39.94 | -0.18 | .86 |
|  | Consistent titles^3^ | 0.03 | 0.05 | 121.00 | 0.53 | .60 |
|  | Inconsistent titles^3^ | -0.01 | -0.01 | 121.20 | -0.10 | .92 |
|  | Image surface^5^ | 0.40 | 0.12 | 36.93 | 1.45 | .15 |
|  | Number of fixations | 0.04 | 0.35 | 4177.00 | 24.19 | < .001 |
|  | Semantic violations^2^ * consistent titles^3^ | -0.01 | -0.02 | 104.80 | -0.43 | .67 |
|  | Semantic violations^2^ * Inconsistent titles^3^ | 0.02 | 0.04 | 105.30 | 0.77 | .45 |
| Liking | Intercept | 4.33 | 0..00 | 72.45 | 39.93 | < .001 |
|  | Dynamic entropy^5^ | -0.14 | -0.02 | 195.20 | -1.01 | .31 |
|  | Stationary entropy^5^ | -0.18 | -0.05 | 311.00 | -2.49 | .01 |
|  | Number of fixations^5^ | 0.02 | 0.04 | 3597.00 | 2.47 | .01 |
| Understanding | Intercept | 4.69 | -0.00 | 68.81 | 29.19 | < .001 |
|  | Dynamic entropy^5^ | 0.19 | 0.02 | 251.30 | 1.52 | .13 |
|  | Stationary entropy^5^ | -0.02 | -0.00 | 3927.00 | -0.28 | .78 |
|  | Number of fixations^5^ | -0.01 | -0.01 | 4341.00 | -0.70 | .48 |
|  |  |  |  |  |  |  |

^1^ Log-transformed; ^2^ Reference category: violations ; ^3^ Reference category: untitled;

^4^ Reference category: consistent; ^5^ centred
